# The Association of microRNA-34a With High Incidence and Metastasis of Lung Cancer in Gejiu and Xuanwei Yunnan

**DOI:** 10.3389/fonc.2021.619346

**Published:** 2021-03-16

**Authors:** Yan Chen, Chun Hou, Liu-xin Zhao, Qiu-chen Cai, Ying Zhang, Da-lun Li, Yao Tang, Hong-yu Liu, Yun-yi Liu, Yue-yan Zhang, Ya-kun Yang, Cheng-wei Gao, Qian Yao, Qi-shun Zhu, Chuan-hai Cao

**Affiliations:** ^1^ School of Life Sciences, Yunnan University, Kunming, China; ^2^ Yunnan Cancer Center, Yunnan Cancer Hospital, The Third Affiliated Hospital of Kunming Medical University, Kunming, China; ^3^ Key Laboratory of the University in Yunnan Province for International Cooperation in Intercellular Communications and Regulations, Yunnan University, Kunming, China; ^4^ School of Chemical Science and Technology, Yunnan University, Kunming, China; ^5^ Taneja College of Pharmacy, University of South Florida, Tampa, FL, United States

**Keywords:** miR-34a, lung cancer, growth, metastasis, CDK6, PTEN, YY1

## Abstract

The incidence and associated mortality of lung cancer in tin miners in Gejiu County and farmers in Xuanwei Country, Yunnan Province have been very high in the world. Current published literatures on the molecular mechanisms of lung cancer initiation and progression in Gejiu and Xuanwei County are still controversial. Studies confirmed that microRNA-34a (miR-34a) functioned as a vital tumor suppressor in tumorigenesis and progression. However, the role and precise mechanisms of miR-34a and its regulatory gene network in initiation and progression of lung cancer in Gejiu and Xuanwei County, Yunnan Province, have not been elucidated. In the current study, we first found that miR-34a was downregulated in Gejiu lung squamous carcinoma YTMLC-90, Xuanwei lung adenocarcinoma XWLC-05, and other non-small cell lung carcinoma (NSCLC) cell lines, and miR-34a overexpression inhibited cell proliferation, migration and invasion, as well as induced cell apoptosis in YTMLC-90 and XWLC-05 cells. Our findings revealed that miR-34a is critical and cannot be considered as the area-specific non-coding RNA in initiation and progression of lung cancer in Gejiu and Xuanwei County. Next we revealed that miR-34a overexpression suppressed lung cancer growth and metastasis partially *via* increasing PTEN but reducing CDK6 expression that might lead to subsequent inactivation of PI3K/AKT pathway. Furthermore, our findings demonstrated that YY1 functioned as a tumor suppressor gene in initiation and progression of lung cancer in Gejiu and Xuanwei County. In conclusion, our findings in the study confirmed that miR-34a overexpression could simultaneously suppress tumor growth and metastasis and play a vital role in tumorigenesis and progression of NSCLC *via* increasing PTEN and YY1 expression, but decreasing CDK6. Most interestingly, our findings also raised doubts about the current ideas about these area-specific diseases.

## Introduction

Statistical data demonstrated that lung cancer is the most prevalent cancer type of cancer as well as the greatest threat to human health and life (1). The incidence and mortality rates of lung cancer in Gejiu and Xuanwei Country are the highest in China (2–4). Since the 1960s, lung cancer in Gejiu and Xuanwei Country has become increasingly attractive for many researchers, and numerous epidemiological and etiological studies have been performed. Previous etiological and epidemiological studies have confirmed that lung cancer in Gejiu and Xuanwei County has its own unique characteristics (2, 5–9). First, high incidence and mortality of lung cancer in the Gejiu Country are related with radon and mine dusts in the air of the workplace, and high incidence and mortality of lung cancer in Xuanwei County was related with PAHs and indoor coal-fired particle. Second, lung squamous cell carcinoma is the most common type of job-related lung cancer for tin miners in Gejiu Country, Yunnan province (10, 11), and lung adenocarcinoma is the most common type of lung cancer for farmers in Xuanwei Country (12). Third, unlike other regions, the incidence of lung adenocarcinoma in non-smoking women in Xuanwei Country was very high, one of the highest in the world (2, 12). And the main clinical features of lung adenocarcinoma in Xuanwei Country were younger onset, high metastasis rate and insensitivity to conventional chemotherapy and radiotherapy (9). Although current epidemiological and etiological findings indicate that genetic variation might play an important role in tumorigenesis and progression of lung cancer in Gejiu and Xuanwei Country, there are few studies on unique genes in Gejiu and Xuanwei lung cancer initiation and progression. Due to high incidence and associated mortality of lung cancer in Gejiu and Xuanwei Country, Mao BL et al. established the YTMLC-90 cell line using tumor issues from a 79-year-old male patient who was a tin miner in Gejiu Country for 17 years and diagnosed with lung squamous cell carcinoma (T2N0M0) (13). Yan FC et al. also established the XWLC-05 cell line which is derived from a female patient who was a 68-years-old Xuanwei permanent resident and diagnosed with moderately differentiated lung adenocarcinoma (14). Since then, several researchers used YTMLC-90 and XWLC-05 to screen antitumor drug and to clarify the precise molecular mechanism for lung cancer molecular targeted therapy (13, 15–17). However, even with the current published literatures, the molecular mechanisms of lung cancer initiation and progression in Gejiu and Xuanwei County still remain controversial. Despite the tremendous efforts made in treatment in recent years, the 5-year overall survival rate of lung cancer patients is very low (18). Thus, new therapeutic strategies were urgently needed.

The miR-34 family was first found in nematodes and is very conservative in evolution. In mammals, the miR-34 family includes miR-34a, miR-34b, and miR-34c (19). The miR-34a gene is located at 1p36.23 and is important in *p53* gene regulation network as well as the most common miRNA transcriptionally regulated by p53 (19). MiR-34a can also upregulate *p53* by the downregulation of *E2F3*, *SIRT1*, and *c-MYC*, thus forming a forward regulatory loop of *p53*-miR-34a (20, 21). Until recently, the findings highlighted miR-34a is a key tumor suppressive miRNA in the development of multiple types of cancer, including lung cancer (22, 23), colorectal cancer (24), breast cancer (25), liver cancer (26), prostate cancer and lymphoma (27). Moreover, the miR-34a-dependent pathway might have wide-range impacts on the hallmarks of cancer including sustaining proliferative signaling, evading growth suppressors, resisting cell death, activating invasion and metastasis, and reprogramming energy metabolism by silencing regulatory gene expressions, thereby inhibiting tumor growth and metastasis (20, 28–30). The main molecular mechanisms of miR-34a-mediated tumor suppression included, but were not limited to repressing cell proliferation (*c-MYC*, *E2F*, *CDK4*, and *CDK6*), promoting cell apoptosis (*Bcl2*, *SITR1*, and *AXL*), and inhibition of cell invasion and metastasis (*c-MET*, *SNAIL*, *MMP9*, *CD44*, and *NOTCH1*) (29, 31–34). Furthermore, miR-34a could also affect the malignant biological behavior of tumor cells through inactivation of MAPK and PI3K signaling pathways (35, 36). However, the role and precise mechanisms of miR-34a in initiation and progression of lung cancer in Gejiu and Xuanwei County, Yunnan Province remained to be further investigated.

In the present study, we revealed for the first time the expression levels and therapeutic potential of miR-34a in lung cancer in tin miners in Gejiu County and farmers in Xuanwei County. We found that overexpression of miR-34a could suppress cell proliferation, cell migration and invasion, and induce cell apoptosis *via* regulating the levels of *CDK6*, *PTEN*, and *YY1* in YTMLC-90 and XWLC-05 cells. Cyclin-dependent kinase 6 (*CDK6*) plays key roles in lung cancer cell proliferation and apoptosis, as well as being a candidate prognostic biomarker for non-small cell lung cancer (37–40). Phosphatase and tensin homolog on chromosome 10 (*PTEN*) is known as a powerful tumor suppressor and frequently mutated in lung cancer and is the negative regulation of the PI3K/mTOR/AKT oncogenic signaling pathway. Numerous findings confirmed that PTEN is crucial for cell proliferation, invasion and survival, and loss of function of PTEN was frequently observed in many types of cancer (41–43). Yin Yang 1 (*YY1*) (also known as *NF-E1*, *UCRBP*, and *CF1*), is a ubiquitous and multifunctional zinc finger transcription factor and can regulate multiple genes associated with multiple cellular processes including cellular differentiation, DNA repair, autophagy, and cell survival. Previous findings confirmed that *YY1* could act as both oncogene and tumor suppressor gene in breast cancer and lung cancer (44–47). Hence, the role of *YY1* in NSCLC progression remains controversial.

In the current study, we evaluated the underlying roles and mechanisms of miR-34a in lung cancer in tin miners in Gejiu County and farmers in Xuanwei County. We found that the expression levels of miR-34a in tumor tissues are significantly lower than those of paired remote control tissues in NSCLC (data not shown). We also found that NSCLC cell lines such as NCI-H157, XWLC-05, and YTMLC-90 cells express low basal levels of endogenous miR-34a compared with human normal bronchial epithelial BEAS-2B cells. Overexpression of miR-34a suppressed cell proliferation, invasion, migration, and induced cell apoptosis in NSCLC cells. We further explored the regulatory mechanisms between miR-34a and its downstream *CDK6*, *PTEN*, and *YY1* to try to find the specificity and similarity of lung cancer in tin miners in Gejiu County and farmers in Xuanwei County compared to other NSCLC regions. Our findings revealed that miR-34a could be a potential therapeutic target for NSCLC. Furthermore, our study could provide a theoretical basis for lung cancer treatment in high-risk areas worldwide.

## Materials and Methods

### Cell Lines and Strains

Xuanwei lung adenocarcinoma cell line XWLC-05, Gejiu lung squamous carcinoma cell line YTMLC-90, human non-small cell lung cancer cell line NCI-H157, human lung adenocarcinoma cell line A549, and human normal bronchial epithelial cell line BEAS-2B were provided by Yunnan Cancer Hospital (the Third Affiliated Hospital of Kunming Medical University) and confirmed *via* short tandem repeat profiling. The lung cell lines were cultured in RPMI1640 medium. BEAS-2B was cultured in DMEM medium.

### Main Reagents

Fetal bovine serum, RPMI1640, DMEM, Opti-MEM medium, PBS, and 0.25% trypsin were purchased from Gibco. pGCMV/EGFP-hsa-miR-34a and pGCMV/EGFP-hsa-miR-NC plasmids were purchased from GenePharma, China. The oligonucleotides of hsa-miR-34a mimics, miRNA mimic control (miR-NC), hsa-miR-34a inhibitors, and miRNA inhibitor control (inhibitor NC) were purchased from RiboBio (RiboBio Co. Ltd., Guangzhou, Guangdong). The siRNAs targeting CDK6 and scrambled siRNA were synthesized by RiboBio (RiboBio Co. Ltd., Guangzhou, Guangdong). MiR-34a (cat. no. HmiRQP0440) and U6 (cat. no. HmiRQP9001) primers were purchased from GeneCopoeia, China. Lipofectamine 2000 was purchased from Invitrogen. RNAprep Pure Kit, miRcute miRNA First-strand cDNA Synthesis Kit and miRcute miRNA qPCR Kit were purchased from Tiangen Biochemical Technology, China. ITaq™ Universal SYBR^®^ Green Supermix Kit and iScript™ cDNA Synthesis Kit were purchased from Bio-Rad. CDK6 and YY1 antibodies were all purchased from Abcam. PTEN monoclonal antibody was purchased from Proteintech Group. DMSO was purchased from Solarbio Corporation. The insert sequence of miR-34a was 5′-TGCTGTGGCAGTGTCTTAGCTGGTTGTGTTTTGGCCACTGACTGACACAACCAGAAGACACTGCCA-3′. The insert sequence of miR-NC was 5′-AATTCGTTCTCCGAACGTGTCACGTGTTTTGGCCACTGACTGACACGTGACATTCGGAGAAA-3′.

## Cell Culture and Transient Transfection

All lung cancer cells were thawed in 37°C water bath, and cultured in RPMI1640 medium containing 10% FBS in cell culture incubator at 37°C and 5% CO_2_. The passage and medium replacement were carried out according to the established methods (13). XWLC-05, YTMLC-90, and NCI-H157cells were seeded in a six-well cell culture plate. Cells were transiently transfected with pGCMV/EGFP-hsa-miR-34a plasmid or pGCMV/EGFP-hsa-miR-NC plasmid using Lipofectamine 2000 (Invitrogen) according to the manufacturer’s protocol when the cell reached to 70% confluence. Similarly, miR-34a mimics, miR-NC, miR-34a inhibitors, inhibitor NC, CDK6 siRNA, and scrambled siRNA were also transfected *via* Lipofectamine 2000 reagent (Invitrogen, Carlsbad, CA, USA) according to the manufacturer’s instructions when the cell density reached 40–50%. After 6 h of transfection, fresh RPMI 1640 medium containing 10% FBS was used to replace the Opti-MEM medium (Gibco, Grand Island, NY, USA). Then, 48 h posttransfection cells were harvested for real-time quantitative PCR (qPCR), western blot and *in vitro* functional assay.

### RNA Extraction and qPCR

Once cells yielded more than 60% confluence, the cells were digested and centrifuged at 300× g for 5 min to remove the supernatant, then cell pellet was suspended. After, the cells were transferred to 1.5 mL micro-centrifuge tubes and pelletized by centrifugation. Total RNAs were extracted using RNAprep Pure Kit according to the manufactory instructions. Then cDNA synthesis was performed using miRcute miRNA First-strand cDNA Synthesis Kit and qPCR was done using miRcute miRNA qPCR Kit to detect the expression levels of miR-34a. U6 was used as an endogenous control. In addition, cDNA was also synthesized using iScriptTM cDNA Synthesis Kit and qPCR was conducted using iTaqTM Universal SYBR^®^ Green Supermix Kit to detect the mRNA expression levels of *CDK6*, *PTEN*, and *YY1*. *RPS13* was used as an endogenous control. The primer sequences were shown in **Table 1**. Three duplicates were set for each group of samples, and the qPCR data were analyzed by 2^−△△CT^ comparative method.

**Table 1:** Primer sequences for PCR.

Gene	Primer sequence (5′–3′)
*CDK6*	F: TCCAGGATAACGGAGGCT
	R:GCCAAACTGAGCAGAGTCTTC
*PTEN*	F: CCGAAAGGTTTTGCTACCATTCT
	R:AAAATTATTTCCTTTCTGAGCATTCC
*YY-1*	F: GCACAAAGATGTTCAGGGATAA
	R: AAGGGCTTCTCTCCAGTATGA
*RPS13*	F:GTTGCTGTTCGAAAGCATCTTG
	R:AATATCGAGCCAAACGGTGAA

F, Forward primer; R, Reverse primer.

### Cell proliferation Was Detected by MTT or CCK8 Assay

48 h posttransfection, cells were digested by 1 mL 0.25% trypsin-EDTA and then centrifuged at 300× g for 5 min. The supernatant was discarded. The cell pellet was suspended and counted using the hemocytometer. The cells of each group were seeded at 2,000 cells per well in a 96-well cell culture plate with approximately six replicates per condition. Each day, one plate was taken out from the incubator for MTT or CCK8 assay for up to 4 days or more according to the manufactory instructions. In brief, tissue culture medium was removed and 10 µL of MTT or CCK8 solution was added into each well and incubated for 4 h at 37°C and 5% CO_2_. OD value was detected using a metric plate reader (Bio-Rad Laboratories, Hercules, CA, USA) at 490 nm wavelength. Data were analyzed using GraphPad Software.

### Cell Migration Was Measured by a Scratch Assay

48 h after transfection, cells were collected from each group and seeded into a six-well cell culture plate at the density of 5 × 10^5^ cells/mL per well. Two milliliters of complete medium was added to each well and cultured in the incubator. When the cells were upon 100% confluent, the cells were washed two times with 1× PBS, and a 10-µL peptide tip was used to scratch the cell monolayer in a straight line to create a new artificial gap of approximately similar size. The cells were then gently washed with 1× PBS buffer, and then RPMI-1640 culture medium was added. The images were obtained using an inverted microscope in a fixed position, and the image observed at 0 h was recorded. The six-well cell culture plate was placed back to the incubator for continuous culture. Then the picture was taken every 24 h. The mean migration rate of cells in each group was calculated according to the selected three fields in fixed points, and data were analyzed using GraphPad Software.

### Cell Invasion Was Measured by a Trans-Well Invasion Assay

The Matrigel was diluted with seven times serum-free medium and added to the upper chamber in a volume of 60 µL. 48 h after transfection, cells were collected from each group and seeded into the upper chamber with the density of 10,000 cells per well in a final volume of 200 µL using Opti-MEM medium. The culture medium (RPMI1640) containing 20% FBS was added into the lower chamber. After incubation at 37°C in 5% CO_2_ for 24 h, the upper chamber was fixed and stained with 200 µL 0.1% crystal violet solution for 1 h. The chambers were washed with 1× PBS, and the cells at the top of the Matrigel membrane were removed with several Q-tips. These cells stained with crystal violet in each group were counted using an inverted microscope with 4×objective. Statistics were done using GraphPad Software.

### The Cellular Morphology and Cell Apoptosis Were Observed by Transmission Electron Microscopy

48 h after transfection, the morphology and cell apoptosis of XWLC-05 and YTMLC-90 cells were observed using transmission electron microscopy. The cells in each group were digested and centrifuged, and 5 × 10^6^ cells were collected in each group. Supernatant was discarded and cell pellets were collected. Cells were fixed with 2.5% glutaraldehyde solution for 2 h and transferred to new 1.5 mL Eppendorf tubes, and then fixed again for 2 h with 1% osmium. After fixation, cells were washed with 1× PBS for several times. The samples were dehydrated with ethanol solution for 20 min and treated with pure acetone for 20 min. The whole sample was put into the diluted embedding agent (acetone: embedding agent = 1:1), placed at room temperature for 1 h, and then soaked in the pure embedding agent, epoxy resin, for embedding overnight. After 24 h, the sample was fixed on the slicer for ultra-thin slicing. A sheet of wax paper was placed on the plate, and the uranium acetate dye was applied. The sections were inserted into the dye gently, and stained at room temperature. After about 20 min, the sheet was washed with ddH_2_O for one to two times, and then observed and photographed under the transmission electron microscope.

### Cell Apoptosis Was Detected by Flow Cytometry

Cell apoptosis was measured by flow cytometry. 48 h after transfection cells in each group were digested and centrifuged at 300× g for 5 min. Cells were then washed with 1× ice-cold PBS for one to two times, and ice-cold PBS was added to suspend cells. Cells were stained by annexin V/PI as previously described (13). Briefly, Annexin V and PI were added to dye at room temperature for 30 min and then were detected by flow cytometry according to the guidelines.

### Western Blot Analysis

48 h after transfection, cells in each group were washed with 1× ice-cold PBS, then a RIPA buffer (Beyotime, China) with protease inhibitors (Roche) was added to lyse the cells. Protein was collected by centrifugation and quantified using BCA assay. Then SDS-PAGE was conducted to separate proteins, and then proteins were transferred onto polyvinylidene fluoride (PVDF) membrane (Millipore, USA) after electrophoresis. Membranes were blocked with TBST buffer solution containing 5% non-fat milk powder for 1 h, and then incubated at 4°C overnight with dilutions the primary antibodies (anti-PTEN, Proteintech, 60300-1-Ig, 1:1,000; anti-CDK6, Abcam ab124821, 1:2,000; anti-YY1, Abcam, ab109237, 1:2,000) (all diluted with TBST buffer solution). The PVDF membrane was washed with TBST buffer solution for three times and then incubated for 2 h at room temperature with 1:4,000 dilutions (v/v) of the horseradish peroxidase-conjugated secondary antibody (Cell Signaling Technology, USA, 7076) or with 1:2,000 dilutions (v/v) of the horseradish peroxidase-conjugated secondary antibody (Cell Signaling Technology, USA, 7074). GAPDH (Abmart, 1:5,000) was used as a loading control. Protein expression was detected using the enhanced chemiluminescence (ECL-plus) reagents (Millipore, USA). The relative protein levels were analyzed using Image J software (USA).

### Statistical Analysis

All experimental data were imaged with GraphPad Prism7 (GraphPad Software, La Jolla, CA, USA) and Image J. All data were analyzed with GraphPad Prism7. All results representing the average triplicate experiments were expressed as the mean ± standard deviation. Statistical analysis was performed using one-way ANOVA with Dunnett’s *post-hoc* test where all comparisons are against a single control, with Tukey’s *post-hoc* test where all groups are compared to one another. A value of P <0.05 was considered statistically significant.

## Results

### Expression Level of miR-34a Was Reduced in NSCLC Cells

**Figure 1:**
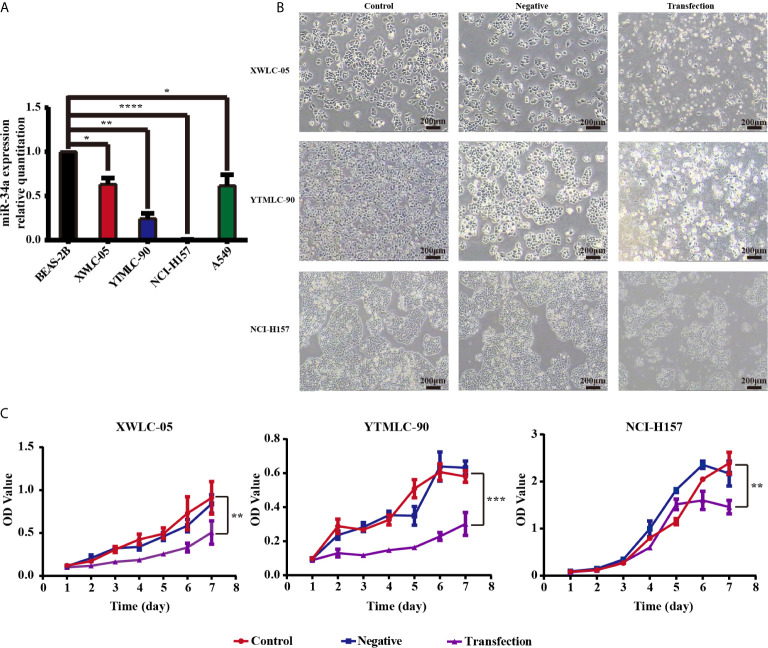
**(A)** The microRNA-34a (miR-34a) expression levels in four types of lung cancer cell lines (XWLC-05, YTMLC-90, NCI-H157, and A549) and a normal lung cell line BEAS-2B were detected by real-time fluorescence quantitative-PCR (qPCR). MiRNA abundance was normalized to U6 as a reference gene, the expression values are normalized to BEAS-2B (n = 3, *P < 0.05, **P < 0.01, ****P < 0.0001, *versus* BEAS-2B). **(B)** The morphology of XWLC-05, YTMLC-90, and NCI-H157 cells was observed at 48 h posttransfection under the optical microscope. The pGCMV/EGFP-hsa-miR-34a plasmid transfection experimental group has remarkable morphologic changes in response to miR-34a overexpression compared with the pGCMV/EGFP-hsa-miR-NC plasmid transfection negative control group and the blank control group. Cell images were captured using 40× magnificaton (scale bar, 200 µm). Transfection, the pGCMV/EGFP-hsa-miR-34a plasmid transfection experimental group. Negative, the pGCMV/EGFP-hsa-miR-NC plasmid transfection negative control group. Control, the blank control group. **(C)** The proliferation rate of lung cancer cells was detected by MTT assay at 48 h posttransfection. The proliferation rate of cells transfected with the pGCMV/EGFP-hsa-miR-34a plasmid was decreased dramatically compared to the pGCMV/EGFP-hsa-miR-NC plasmid transfection negative control group and the blank control group (n = 3, *P < 0.05, **P < 0.01, ***P < 0.001, ****P < 0.0001 respectively *versus* corresponding control).

We used qPCR to assess the expression levels of miR-34a in NSCLC cells. RNAs were extracted fromXWLC-05, YTMLC-90, NCI-H157, A549, and BEAS-2B cells, and OD (260/280) values were measured which were all greater than 1.90. Electrophoresis showed that RNA bands were clear and bright with good integrity and no degradation, which could be used as the template for qPCR. Three repeated qPCR results showed that NCI-H157, XWLC-05, YTMLC-90, and A549 cells expressed low basal levels of endogenous miR-34a compared with BEAS-2B cells (*P < 0.05, **P < 0.01 and ****P < 0.0001 respectively) (**Figure 1A**).

### The Upregulation of miR-34a Could Inhibit the Growth and Proliferation of XWLC-05, YTMLC-90, and NCI-H157 Cells

Previous finding demonstrated that miR-34a has been shown to possess tumor-suppressive functions in NSCLC cell lines such as A549 (p53 wild-type), H460 (p53 wild-type), and H1299 (p53 mutant) (48). In particular, we were interested in the role of miR-34a in Xuanwei and Gejiu NSCLC in the present study. To test whether expressing exogenous miR-34a could impact on the proliferation of lung cancer cells in Xuanwei and Gejiu, China, XWLC-05, YTMLC-90, and NCI-H157 cells were transiently transfected with the pGCMV/EGFP-hsa-miR-34a or pGCMV/EGFP-hsa-miR-NC plasmid and cell proliferation was measured by MTT at 48 h posttransfection. At 48 h after being transfected, marked morphology changes were observed in the cells transfected with the pGCMV/EGFP-hsa-miR-34a plasmid using light microscopy, for example, increased detached cells, increased intercellular space and impurities, when compared to the cells transfected with the pGCMV/EGFP-hsa-miR-NC plasmid. The morphology of the cells between the pGCMV/EGFP-hsa-miR-NC plasmid transfection negative control group and the blank control group has no obvious changes (**Figure 1B**). Interestingly, there were no obvious morphologic changes in XWLC-05, YTMLC-90, and NCI-H157 cells transfected with pGCMV/EGFP-hsa-miR-34a.

Furthermore, the rate of cell proliferation in the pGCMV/EGFP-hsa-miR-34a plasmid transfection experimental group was significantly lower than that of the blank group or the negative control group at 48 h posttransfection (**P < 0.01, ***P < 0.001 respectively) (**Figure 1C**). Our data demonstrated that overexpression of miR-34a inhibited XWLC-05 and YTMLC-90 cell proliferation.

### MiR-34a Upregulation in XWLC-05, YTMLC-90, and NCI-H157 Cells Could Inhibit Cell Migration and Invasion

**Figure 2:**
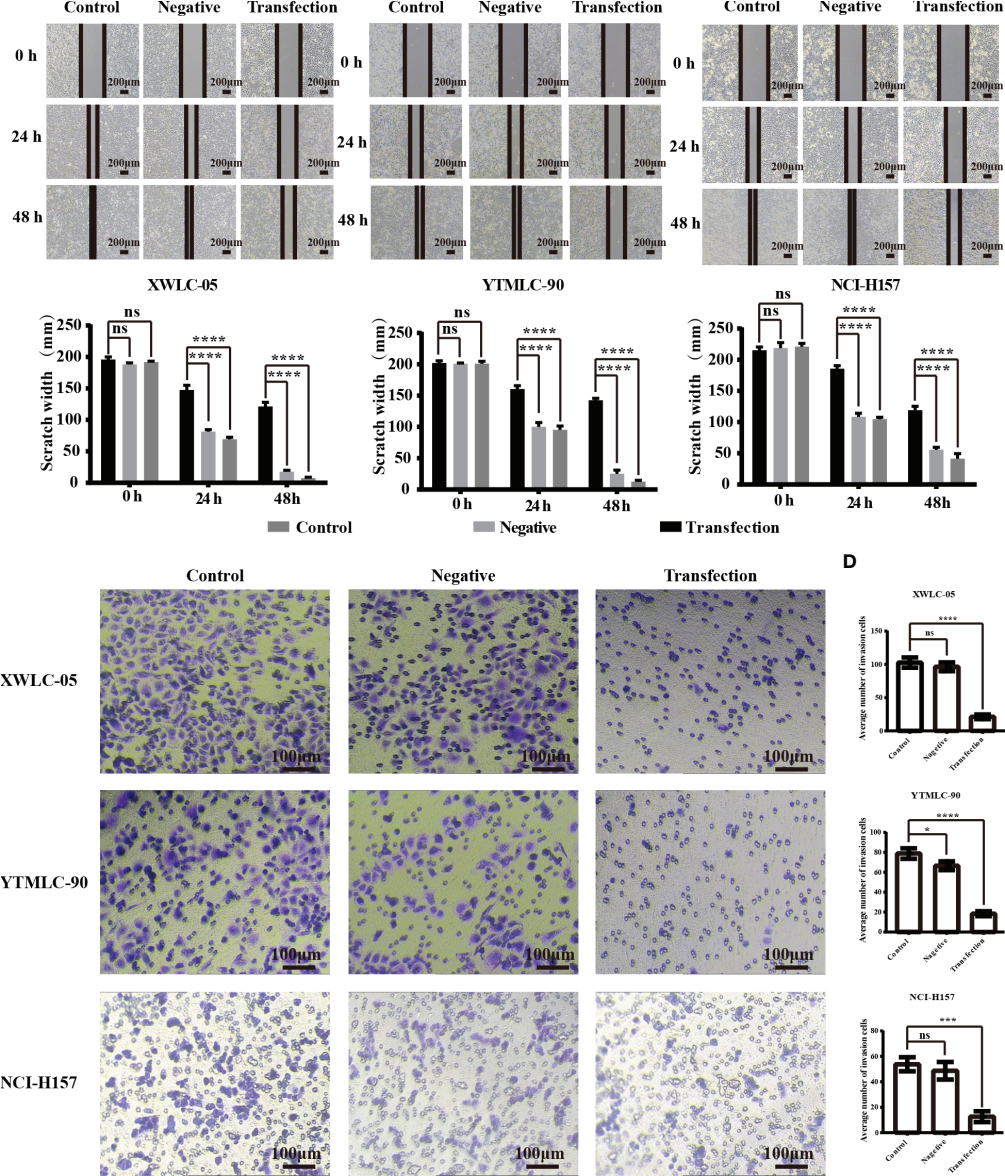
**(A)** The cell migration ability of lung cancer cells (XWLC-05, YTMLC-90, and NCI-H157) after miR-34a up-regulation was detected by wound healing assay. Representative images of wound healing area in each group under a light microscope (magnification, x40; scale bar, 200 µm); **(B)** Histograms indicated the migration rate of each group at 24 and 48 h after transfection. Cell migration was detected using the wound healing assays. Uniform scratches were made in XWLC-05, YTMLC-90, and NCI-H157 cells and the serial photographs were obtained at different time points posttransfection. The scratch width for the cells transfected with pGCMV/EGFP-hsa-miR-34a plasmid was decreased dramatically compared to the pGCMV/EGFP-hsa-miR-NC plasmid transfection negative control group and the blank control group (n = 3, ****P < 0.0001 respectively *versus* the corresponding control). **(C)** The cell invasion ability of lung cancer cells (XWLC-05, YTMLC-90, and NCI-H157) after miR-34a up-regulation was detected by the trans-well invasion assay. Representative images of the invasive cells in each group under a light microscope (magnification, ×100; scale bar, 100 µm). The invasive cells were stained with 0.1% crystal violet and appeared in purple; **(D)** Histograms indicated the invasion rate of each group at 48 h after transfection. The average number of the invasive cells was calculated from five random views. The average number of the invasive cells in the pGCMV/EGFP-hsa-miR-34a plasmid group was decreased dramatically compared to the pGCMV/EGFP-hsa-miR-NC plasmid transfection negative group and the blank control group (n = 3, *p < 0.05, ***P < 0.001, ****P < 0.0001 respectively *versus* the corresponding control). ns, statistical significance exist.

We used the wound-healing assay to investigate the influence of expressing exogenous miR-34a on migration of lung cancer cells in Xuanwei and Gejiu, China. Under the light microscope, each group of cells was observed at 0, 24, and 48 h and then photographed. The transfection experimental group, the blank control group, and the negative control group at 0 h have almost the same scratch width. However, there was a marked difference at 24 h among them. The scratch width for the negative control group and the blank control group reduced rapidly, but the scratch width for the experimental group was still clearly visible. Moreover, no gap existed at all in the blank control group and the negative control group at 48 h while the scratch for the experimental group was still clearly visible. Those results indicated that miR-34a overexpression in XWLC-05, YTMLC-90, and NCI-H157cells could significantly weaken cell migration (**Figures 2A, B**). The differences between the experimental group and the control groups have statistical significance and all the P values were less than 0.001 (****P < 0.0001 respectively). No statistically significant difference was found between the blank group and the negative control group.

Next, we used the transwell invasion assay to determine the effect of expressing exogenous miR-34a on invasion of lung cancer cells in Xuanwei and Gejiu, China. As shown in **Figures 2C, D** the average number of the cells that penetrated the Martrigel in the XWLC-05 transfection experimental group (21.20 ± 3.53) was significantly higher than the negative control group (96.40 ± 6.57) and the blank group (102.70 ± 7.66) (****P<0.0001 respectively). Meanwhile, the average number of the invasive cells in the YTMLC-90 transfection experimental group (18.40 ± 2.45) was significantly higher than the negative control group (66.30 ± 4.77) and the blank group (78.80 ± 5.23) (****P < 0.0001 respectively). The average number of the invasive cells in the NCI-H157 transfection experimental group (12.60 ± 4.22) was also significantly higher than that in the negative control group (48.70 ± 6.89) and the blank control group (53.80 ± 5.47) (***P < 0.001 respectively).

### The Upregulation Expression of miR-34a Could Induce the Apoptosis of XWLC-05 and YTMLC-90 Cells

**Figure 3:**
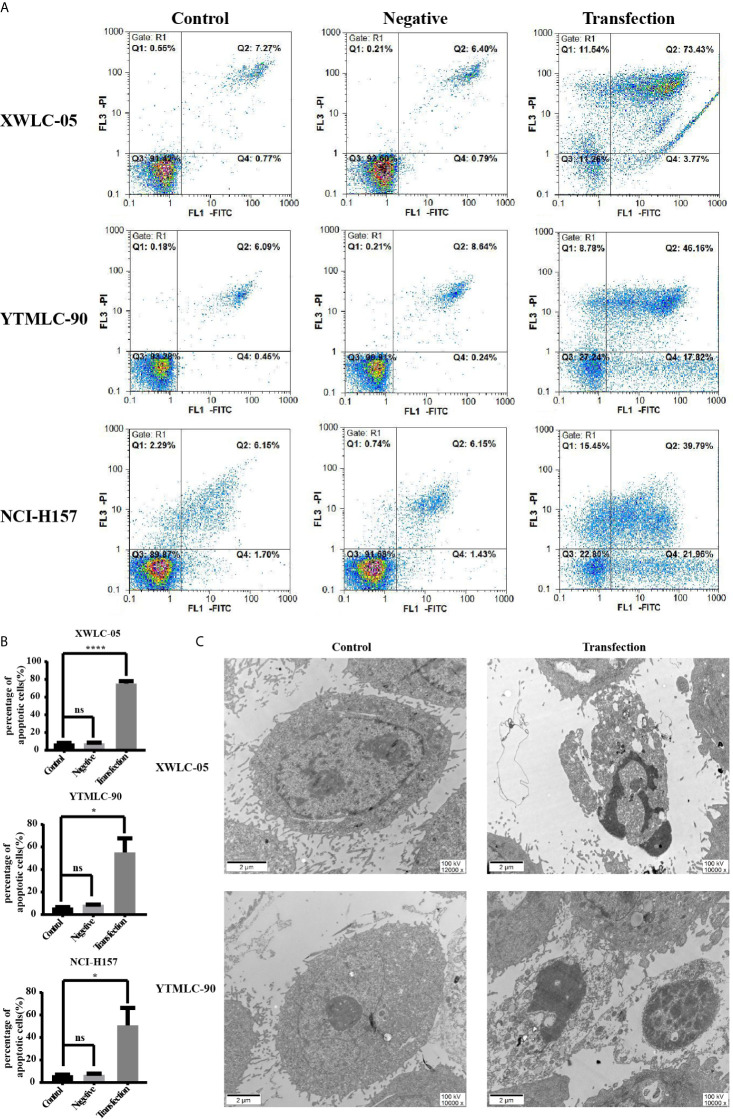
Apoptosis of cells in each group at a qualitative level. **(A)** Flow cytometry analysis with Annexin V-PI staining was performed to evaluate the percentage of apoptotic cells in each group. **(B)** Histograms indicated the percentage of apoptotic cells in each group at 48 h after transfection. The percentage of apoptotic cells in the transfection experimental group was significantly increased compared with that of the controls. Data represented mean ± SD of three independent repeats (n = 3, *P < 0.05, ****P < 0.0001 respectively *vs.* the bland control and the negative control). ns, statistical significance exist. **(C)** Overexpression of miR-34a in XWLC-05 and YTMLC-90 cells could cause obvious characteristic features of apoptosis at 48 h posttransfection. The images were taken under the transmission electron microscope (magnifications: 10,000× and 12,000×, scale bar 2μm). Transfection, the pGCMV/EGFP-hsa-miR-34a plasmid transfection experimental group. Control, the blank control group.

In order to assess whether expressing exogenous miR-34a could induce the apoptosis of lung cancer cells in Xuanwei and Gejiu, China, we transfected XWLC-05 and YTMLC-90 cells with the pGCMV/EGFP-hsa-miR-34a or pGCMV/EGFP-hsa-miR-NC plasmid. The cells of each group were collected at 48 h posttransfection and then electron microscopy analysis was performed according to the protocol. The apoptosis rate in the transfection experimental group was significantly elevated compared to the negative control group and the blank control group (*P < 0.05, ****P < 0.0001 respectively) (**Figures 3A, B**). As shown in **Figure 3C**, the cells in the transfection experimental group presented obvious characteristic features of apoptosis, such as chromatin coagulation and fragmentation, the decreased cytoplasm and the increased density, as well as the apoptotic bodies. Our data suggested that the up-regulation of miR-34a in XWLC-05 and YTMLC-90 could induce the apoptosis of transfected cells.

### The Expression of PTEN, CDK6, and YY1 at mRNA and Protein Levels Could Be Influenced by miR-34a Overexpression in NSCLC Cells

**Figure 4:**
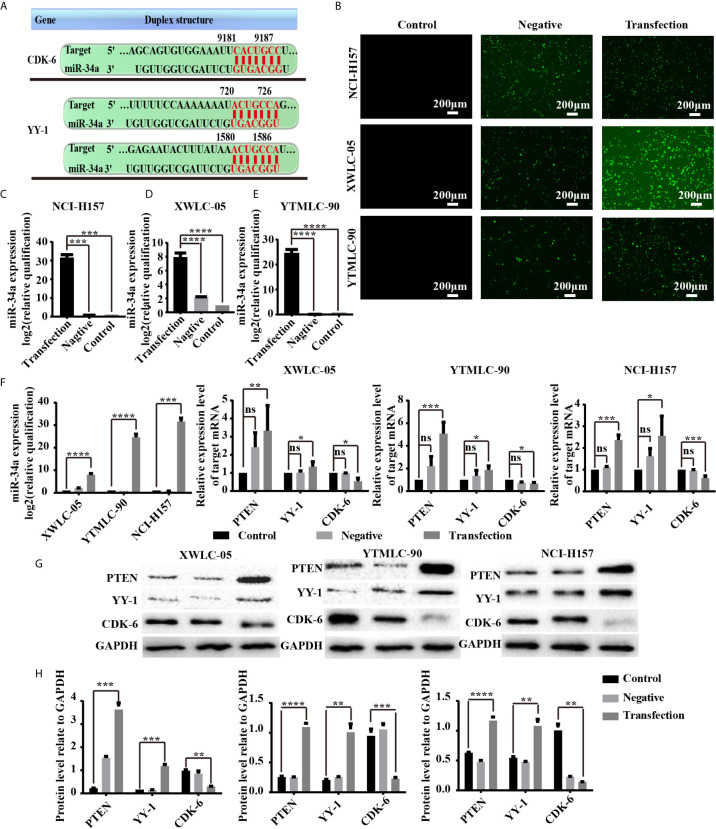
**(A)** miR-34a potential target genes and cell transfection efficiency was determined. The 3′-UTR of YY1 and CDK6 mRNA are potential targets for miR-34a and the seed matching sequences are marked in red. The miRNA targets were predicted by Target Scan Human Release 6.2 (http://www.targetscan.org). **(B)** The expression levels of enhanced green fluorescent protein (EGFP) transfected with the pGCMV/EGFP-hsa-miR-34a or pGCMV/EGFP-hsa-miR-NC for 48 h were observed using a fluorescent inverted microscope. **(C–E)** The expression levels of miR-34a were measured by qPCR at 48 h posttransfection shown in the bar graphs. miRNA abundance was normalized to U6 as a reference gene (n = 3, ***P < 0.001, ****P < 0.0001 respectively *versus* the corresponding control). NC indicated the negative control (transfected with the pGCMV/EGFP-hsa-miR-NC plasmid), RQ indicated relative quantitation. **(F)** The mRNA expression levels of *PTEN*, *YY-1*, and *CDK6* in lung cancer cells were detected by qPCR at 48 h after transfection. mRNA abundance was normalized to RPS13 (n = 3, *P < 0.05, ***P < 0.001, ****P < 0.0001 respectively *versus* the corresponding control). **(G, H)** PTEN, YY-1, and CDK6 protein levels were determined at 48 h after transfection by western blot assay. The GAPDH levels were also used as a reference protein expression, NC indicated the negative control (transfected with the pGCMV/EGFP-hsa-miR-NC plasmid), and RQ indicated relative quantitation (n = 3, **P < 0.01, ***P < 0.001, ****P < 0.0001 respectively *versus* the corresponding control). ns, statistical significance exist.

Next, we sought to clarify the potential mechanism of miR-34a in lung cancer in Xuanwei and Gejiu, China. First, we used TargetScan 7.2 (http://www.targetscan.org/vert_72/), MiRTarBase (http://mirtarbase.mbc.nctu.edu.tw/php/index.php), and StarBase V3.0 databases (http://starbase.sysu.edu.cn/) to predict possible target genes of miR-34a. The bioinformatic analysis showed that *CDK6* and *YY1* were potential target genes of miR-34a (**Figure 4A**). Sencond, XWLC-05, YTMLC-90, and H157 cells were transfected with pGCMV/EGFP-hsa-miR-34a or pGCMV/EGFP-hsa-miR-NC plasmids to demonstrate whether high expression levels of miR-34a correlated with the expression of *PTEN*, *CDK6*, and *YY1* in lung cancer. Transfection efficiency was assessed by fluorescence quantification of enhanced green fluorescent protein (EGFP). We found that EGFP-positive cells in the transfection experimental group or the negative control group were higher than those in the blank control at 48 h after transfection (**Figure 4B**), which demonstrated higher *EGFP* gene transfection efficiency. To further evaluate the efficacy of miR-34a overexpression in XWLC-05, YTMLC-90, and NCI-H157 cells, the expression level of miR-34a was measured by qPCR at 48 h posttransfection. As shown in **Figures 4C–E**, the expression levels of miR-34a in the transfection experimental group were significantly increased (***P < 0.001, ****P < 0.0001 respectively), but no significant changes were observed between the negative control group and the blank control. Then, *PTEN*, *CDK6*, and *YY1* expression was confirmed by qPCR and western blot at 48 h after transfection. As expected, qPCR results showed that the expression levels of *pten* (**P < 0.01, ***P < 0.001 respectively) and *YY1* (*P < 0.05 respectively) mRNA were significantly elevated in the transfection experimental group compared with the negative control group and the blank group when the expression levels of miR-34a were unregulated, while *CDK6* (*P < 0.05, ***P < 0.001 respectively) mRNA expression levels were just the opposite (**Figure 4F**). Western blot results, also consistent with qPCR assay, showed that PTEN (***P < 0.001, ****P < 0.0001 respectively) and YY-1 (**P < 0.01, ***P < 0.001 respectively) protein expression levels were significantly increased in the transfection experimental group compared with the negative control group and the blank group, while the expression levels of CDK-6 (**P < 0.01, ***P < 0.001 respectively) protein in the experimental group were significantly reduced (**Figures 4G, H**). Meanwhile, XWLC-05 and NCI-H157 cells were also transiently transfected with miR-34a inhibitors, inhibitor NC, miR-34a mimics and miR-NC respectively using Lipofectamine 2000 to further confirm the effects of synthetic miR-34a on the expression of CDK6 and YY1. The same results were also observed (**Supplementary Figure 1**). These results confirmed that enforced overexpression of miR-34a increased the expression of PTEN and YY-1 proteins while inhibiting the expression of CDK-6 protein in XWLC-05, YTMLC-90, and NCI-H157 cells.

Given the critical role of CDK6 in lung cancer progression, XWLC-05 and NCI-H157 cells were co-transfected with CDK6 siRNA and miR-34a to elucidate whether CDK6 is involved in miR-34a-mediated inhibitory effect on lung cancer cell progression in the present study. CDK6 protein expression was confirmed by western blot, and CCK8 assay was performed to detect the viability of the cells co-transfected with si-CDK6 and miR-34a. As shown in **Supplementary Figure 2A**, CDK6 siRNA could suppress the basal endogenous CDK6 protein expression in XWLC-05 and NCI-H157 cells. Importantly, the si-CDK6-transfected lung cancer cells showed a significant reduction in the viability of cells compared to si-NC-transfected or miR-NC-transfected cells. Furthermore, the lung cancer cells co-transfected with si-CDK6 and miR-34a exhibited the lowest viability (*P* < 0.05, *P* < 0.01 respectively) (**Supplementary Figure 2B**). The data are also consistent with our unpublished findings that artemether inhibited the malignant biological behaviors of lung cancer cells portionially *via* enhancing miR-34a expression, as well as reducing CDK6 expression (data not shown). Thus, these findings indicated that miR-34a could suppress the malignant biological behaviors of lung cancer cells partially *via* reducing CDK6 expression.

## Discussion

Data and statistics about cancer incidence and mortality confirmed lung cancer was the top cancer for men and women in China, and especially in Xuanwei and Gejiu Country (1–3). Furthermore, epidemiological and etiological findings demonstrated that lung cancer in Xuanwei and Gejiu Country presented regional difference and professional specificity, which suggested that there might be unique genetic variations in the development of Xuanwei and Gejiu lung cancer (7–9). For example, mutations in MUC16 gene are observed in 50% of lung cancer patients residing in Xuanwei and dysregulation of MUC16 was involved in the progression of Xuanwei lung cancer (49, 50). These findings suggested that air pollution-related genes might be critical in Xuanwei lung cancer progression. Numerous studies were done to elucidate the multiple mechanisms of Xuanwei and Gejiu lung cancer; however, there were currently no effective treatments for those local lung cancer patients, and the precise molecular mechanism involved remained largely unclear (9, 13, 49, 51–54). Previous findings confirmed that microRNAs play critical roles in the development of Xuanwei and Gejiu lung cancer and might be diagnostic biomarkers (9, 13, 55). In the current study, we demonstrated for the first time the miR-34a function and the miR-34a-related regulatory mechanisms in the tumorigenesis and progression of lung cancer in Xuanwei and Gejiu Country, Yunnan Province, China.

Dysregulation of miR-34a expression was found in multiple cancers including lung cancer and correlated with poor prognosis (22, 28, 56). We mainly focused on the role of miR-34a in Xuanwei and Gejiu NSCLC in the current study. Consistent with previous findings (22, 56), miR-34a was significantly reduced in Xuanwei lung cancer tissues (data not shown) and cell lines. In contrast, Pan HL et al. performed microRNA microarray analysis to identify abnormal miRNAs critical for Xuanwei NSCLC tumorigenesis in six Xuanwei NSCLC and four NSCLC tissues from control regions where smoky coal was not used and showed that miR-34a was not found to change significantly (55). A possible reason for this discrepancy is that microRNA microarray analysis was based on a small number of cases. Next, XWLC-05, YTMLC-90, and NCI-H157 cells were transiently transfected with the pGCMV/EGFP-hsa-miR-34a or pGCMV/EGFP-hsa-miR-NC plasmid using Lipofectamine 2000 to further demonstrate whether miR-34a functions as a tumor suppressor in lung cancer in Gejiu and Xuanwei County based on the previous finding that miR-34a has been shown to possess tumor-suppressive functions in NSCLC cell lines such as A549 (p53 wild-type), H460 (p53 wild-type), and H1299 (p53 mutant) (48). Our findings in the study confirmed that vector-mediated miR-34a overexpression inhibited cell proliferation, migration, and invasion, and induced cell apoptosis in XWLC-05 and YTMLC-90 cells, and indicated that miR-34a acts also as a tumor suppressor and is critical for the tumorigenesis and progression of lung cancer in Xuanwei and Gejiu. Future work will also need to confirm the function of miR-34a in XWLC-05 and YTMLC-90 cells by using a miR-34a mimicker and miR-34a antagonist. Furthermore, we tried to find out the key genes in the tumorigenesis and progression of Xuanwei and Gejiu NSCLC regulated by miR-34a in the current study. Previous findings confirmed that CDK6 was critical in the multiple-step tumorigenesis (38, 39). Moreover, miR-34a could repress CDK6 protein expression in different cancer cells suppressing cell proliferation and promoting apoptosis in nasopharyngeal cancer (57), cervical cancer (58) and glioblastoma (59). It has also been shown that miR-34a expression was inversely correlated with CDK6 expression in NSCLC (23). What is more, overexpression of miR-34 could reduce the expression levels of MET and CDK6 which have interaction with PI3K/AKT signaling in cancer cells (60–62). In order to further determine whether miR-34a overexpression could inhibit cell proliferation and cause cell apoptosis *via* suppressing *CDK6* gene expression in Xuanwei and Gejiu lung cancer, the protein and mRNA expression levels of *CDK6* were examined in XWLC-05 and YTMLC-90 cells following vector-mediated miR-34a transfection. Our results in the study showed that CDK6 expression was significantly reduced in XWLC-05 and YTMLC-90 cells transfected with the pGCMV/EGFP-hsa-miR-34a plasmid (23). Similar results were also observed in the miR-34a mimics-transfected XWLC-05 cells. Meanwhile, silencing of CDK6 has a synergic influence on the function of miR-34a in XWLC-05 cells. Our findings reveal that miR-34a negatively regulates CDK6 expression and suppresses the malignant biological behaviors of lung cancer cells, which were consistent with the previous findings (23). Studies have also verified that enhanced miR-204 (63), miR-641 (64), miR-377 (65) or miR-137 (66) could suppress *CDK6* expression in lung cancer. These findings suggested that multiple major miRNAs contributed to *CDK6* expression in lung cancer. Next, previous findings demonstrated that overexpression of miR-34a possesses tumor-suppressive functions *via* upregulating PTEN expression that acts as a critical negative regulator of PI3K/mTOR/AKT pathway in cancer cells (67, 68). In agreement with these findings, the current study demonstrated that miR-34a overexpression could remarkably increase the expression levels of tumor suppressor gene *PTEN* in Xuanwei and Gejiu lung cancer. Of note, PTEN has been confirmed to have great influence on aggressive phenotypes of NSCLC cells (68, 69). Loss of PTEN caused activation of the PI3K/AKT pathway and leading to lung cancer invasion and metastasis (70, 71). The findings of the present study indicated that vector-mediated miR-34a overexpression reduced invasion and metastasis of Xuanwei and Gejiu lung cancer partially by increasing PTEN expression levels which resulted in PI3K/AKT pathway inactivation. Future works will need to elucidate the more precise regulatory mechanism between the dysregulation of miR-34a and PTEN expression. Besides, our current study verified the regulatory mechanism between miR-34a and YY1 expression in repressing NSCLC tumor growth and metastasis. It has been reported that *YY1* was a direct target gene of miR-34a, and enhanced miR-34a expression could reduce cell proliferation, migration and invasion partially by suppressing YY1 expression in glioblastoma multiforme (72), liver cancer (73) and esophageal squamous cell carcinoma (74). However, our present findings showed that miR-34a overexpression led to enhanced YY1 expression at both mRNA and protein level in Xuanwei and Gejiu lung cancer. The role and mechanisms of *YY1* gene in tumorigenesis and development remain controversial (47). For example, previous research showed that *YY1* acted as a tumor suppressor gene in lung cancer (75). However, some research confirmed that *YY1* served an oncogene role in the occurrence and development of lung cancer (76). Our data suggested that *YY1* functioned as a tumor suppressor gene and miR-34a blocked lung cancer progression partly *via* increasing YY1 expression. However, future work will also need to reduce or increase the exogenous expression levels of PTEN and YY1 in cell lines and investigate miR-34a expression under these conditions to better understanding of the interaction between these genes and miR-34a. Interestingly, the present study did not observe any significant change in the miR-34a function and the miR-34a-related regulatory mechanisms in the initiation and progression of NSCLC in Xuanwei and Gejiu County compared to other NSCLC in control regions. Thus, our present study have also highlighted that the dysregulation of miR-34a was also essential for the progression of Xuanwei and Gejiu NSCLC. More importantly, our present and previous studies suggested that dysregulation of key miRNAs was the common events both in the progression of the area-specific NSCLC and other NSCLC in control regions (13). However, future studies are required on the precise regulatory network involved in the downstream molecules of PTEN, YY1, and CDK6 that could contribute to miR-34a-mediated tumor suppression for the clinical treatment of lung cancer in Gejiu and Xuanwei County, Yunnan Province.

**Figure 5:**
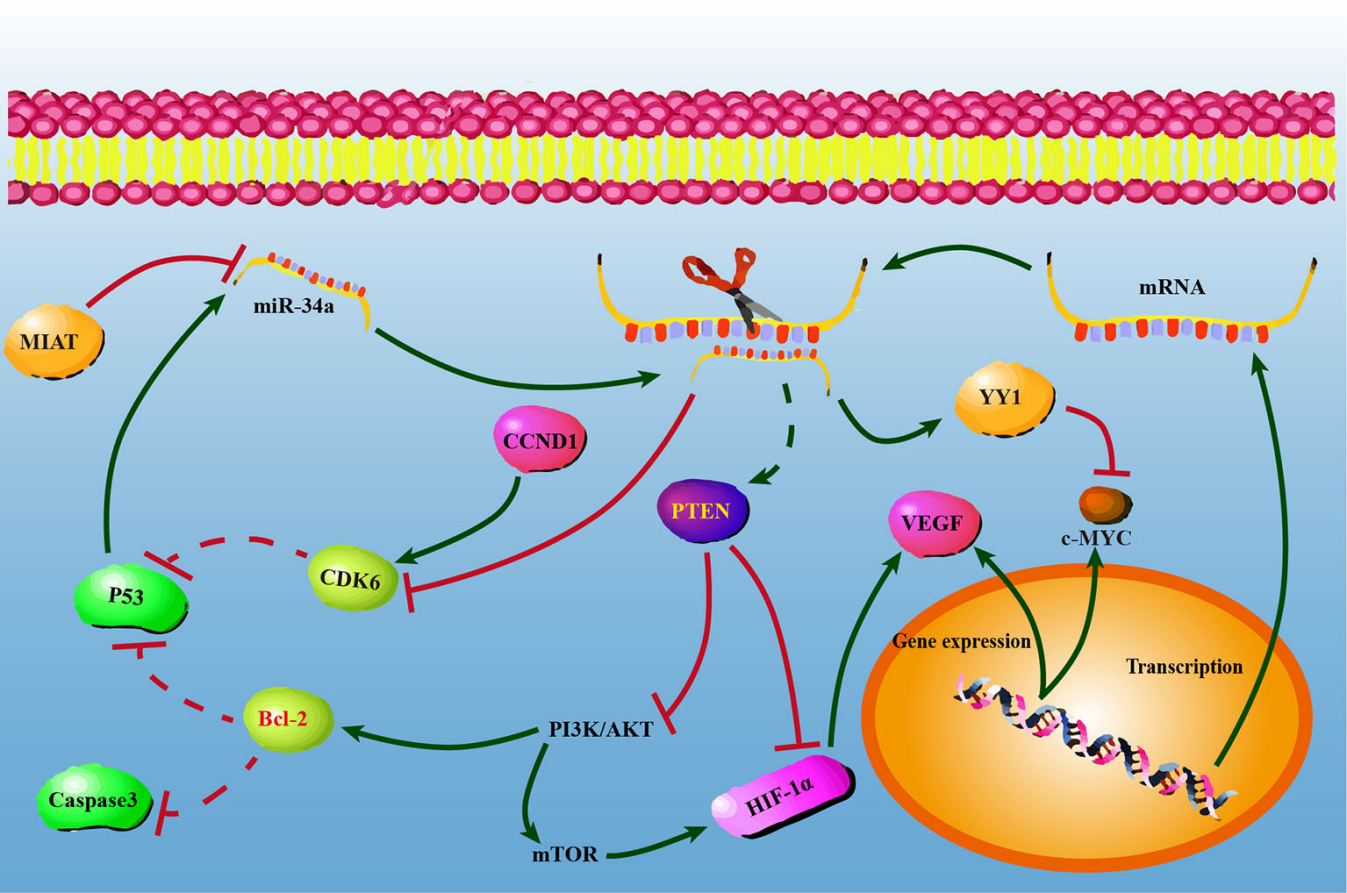
The possible regulatory mechanisms between miR-34a and *PTEN/YY1/CDK6* in non-small lung cell carcinoma cells. At the post-transcriptional level, *PTEN/YY1/CDK6* expression levels were regulated by miR-34a. Overexpression of miR-34a activated PTEN and YY-1, while repressed CDK6 at the mRNA and protein levels. PTEN, YY1, and CDK6 may have a single or combined effect on NSCLC cell proliferation, migration, invasion, and apoptosis.

In conclusion, our present study confirmed that miR-34a overexpression could suppress NSCLC progression by upregulating PTEN and YY1 expression while downregulating CDK6 expression, and indicated that PI3K/AKT signaling might be involved in miR-34a-regulatory mechanisms. It’s worth to investigate whether miR-34a overexpression suppresses tumor growth and metastasis by inhibiting PI3K/AKT signaling. The possible regulatory mechanisms of miR-34a were shown in **Figure 5**. Our current findings further underline the pivotal roles played by miR-34a, facilitate a better understanding of miR-34a in the initiation and progression of NSCLC, as well as suggest that miR-34a may have potential therapeutic value for NSCLC. Therefore, future studies will be required to elucidate the precise mechanisms involved in the downstream molecules of PTEN, YY1 and CDK6 that could contribute to the miR-34a-mediated suppression of lung cancer progression. Besides, a prior study showed that aberrant expression of miR-34a in normal cells or massive necrotic cell death was observed in miR-34a treated tumors (28, 69). Thus, future work will need to investigate genotoxic effects of miR-34a overexpression in human normal bronchial epithelial cells BEAS-2B and in animals. Notably, our findings also demonstrated that miR-34a is critical and cannot be considered as the area-specific non-coding RNA in lung cancer progression in Gejiu and Xuanwei County, and suggested that dysregulation of key miRNAs was common event in the progression of Xuanwei and Gejiu NSCLC. It is hard to say whether the high incidence of Xuanwei and Gejiu NSCLC in South China could only be attributed to scale-specific effects of environmental variables in the area or specific molecular genetic variation. Meanwhile, our findings also suggested that cancer cells might share similar regulatory mechanisms with PTEN, YY1, and CDK6 controlled by miR-34a.

## Data Availability Statement

The data that support the findings of this study are openly available in Database of Differentially Expressed Proteins in Human Cancer at https://doi.org/10.25504/FAIRsharing.dzr6rp.

## Author Contributions

Conceived and designed the experiments: ZQ-S, YC, C-HC. Performed the experiments: L-XZ, YC, YZ, D-LL, YT, H-YL, Y-YL, Y-YZ, Y-KY. Analyzed the data: Q-SZ, C-HC, YC, L-XZ. Contributed reagents/materials/analysis tools: YC, ZQ-S, C-WG. Wrote the paper: YC, ZQ-S, C-HC, Q-CC, CH. All authors contributed to the article and approved the submitted version.

## Funding

The present study was supported by grants from the National Natural Science Foundation of China (grant no. 81560380), Yunnan Medical Discipline Leader Project (grant no. D-201601), the Project of Medical and Health Technology Development Program of Yunnan Province (grant no. 2017NS203), and Fund Project of School of Life Sciences, Yunnan University (grant no. S020054 and S030011)

National Natural Science Foundation of China, grant/award number: 81560380; the Academic Leaders Training Program of Yunnan Provincial Health and Family Planning Commission, grant/award number: D-201601; The Project of Medical and Health Technology Development Program of Yunnan Province, Grant/Award Number: 2017NS203; Fund Project of School of Life Sciences, Yunnan University, grant/award number: S020054 and S030011.

## Conflict of Interest

The authors declare that the research was conducted in the absence of any commercial or financial relationships that could be construed as a potential conflict of interest.

## Supplementary Material

Supplementary Figure 1 Synthetic miR-34a negatively regulates the expression levels of CDK6 and YY1 in lung cancer cells. **(A)** XWLC-05 and NCI-H157 cells were transfected with miR-34a inhibitors, inhibitor NC, miR-34a mimics, or miR-NC using Lipofectamine 2000 (Invitrogen). Quantitative PCR (qPCR) assay was performed to measure the mRNA expression levels of CDK6 and YY1 in the transfected lung cancer cells. RPS13 was used as the internal control. **(B)** The levels of CDK6 protein expression in the transfected lung cancer cells were measured by western blot. GAPDH was used as the internal control.

Supplementary Figure 2 CDK6 is involved in miR-34a-mediated inhibitory effect on lung cancer cell growth. **(A)** XWLC-05 and NCI-H157 cells were transfected with CDK6 siRNA, or scramble siRNA at a final concentration of 50 nM using Lipofectamine 2000 (Invitrogen). The levels of CDK6 protein expression were measured by western blot. GAPDH was used as the internal control. Blank: XWLC-05 and NCI-H157 cells; Negative: XWLC-05 and NCI-H157 cells transfected with scramble siRNA. **(B)** CCK8 assay was used to detect the viability of the cells co-transfected with si-CDK6 and miR-34a.
