# Supplementary figures and images for: The Association of microRNA-34a With High Incidence and Metastasis of Lung Cancer in Gejiu and Xuanwei Yunnan
Source: Front Oncol. 2021 Mar 16;11:619346. doi: 10.3389/fonc.2021.619346 (PMC8008071; doi:10.3389/fonc.2021.619346)

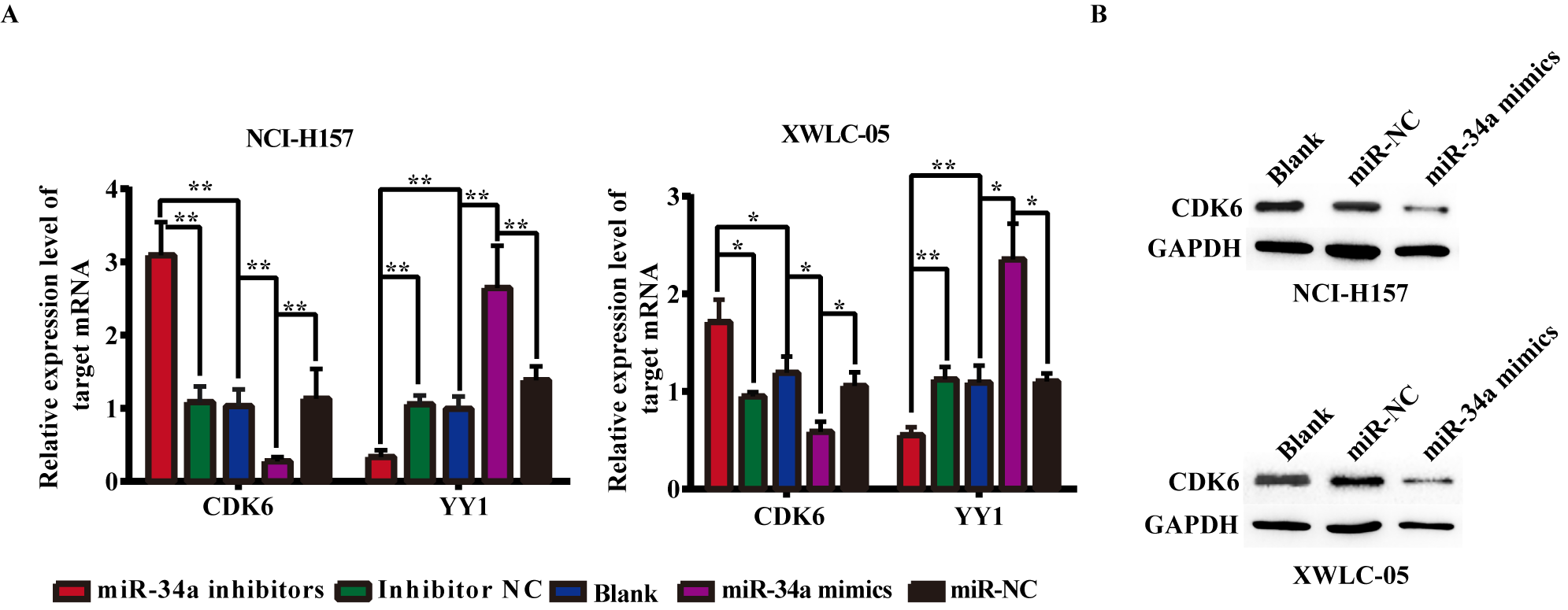

Supplement: Supplementary Figure 1 — Synthetic miR-34a negatively regulates the expression levels of CDK6 and YY1 in lung cancer cells. (A) XWLC-05 and NCI-H157 cells were transfected with miR-34a inhibitors, inhibitor NC, miR-34a mimics, or miR-NC using Lipofectamine 2000 (Invitrogen). Quantitative PCR (qPCR) assay was performed to measure the mRNA expression levels of CDK6 and YY1 in the transfected lung cancer cells. RPS13 was used as the internal control. (B) The levels of CDK6 protein expression in the transfected lung cancer cells were measured by western blot. GAPDH was used as the internal control. [file Image_1.tif]

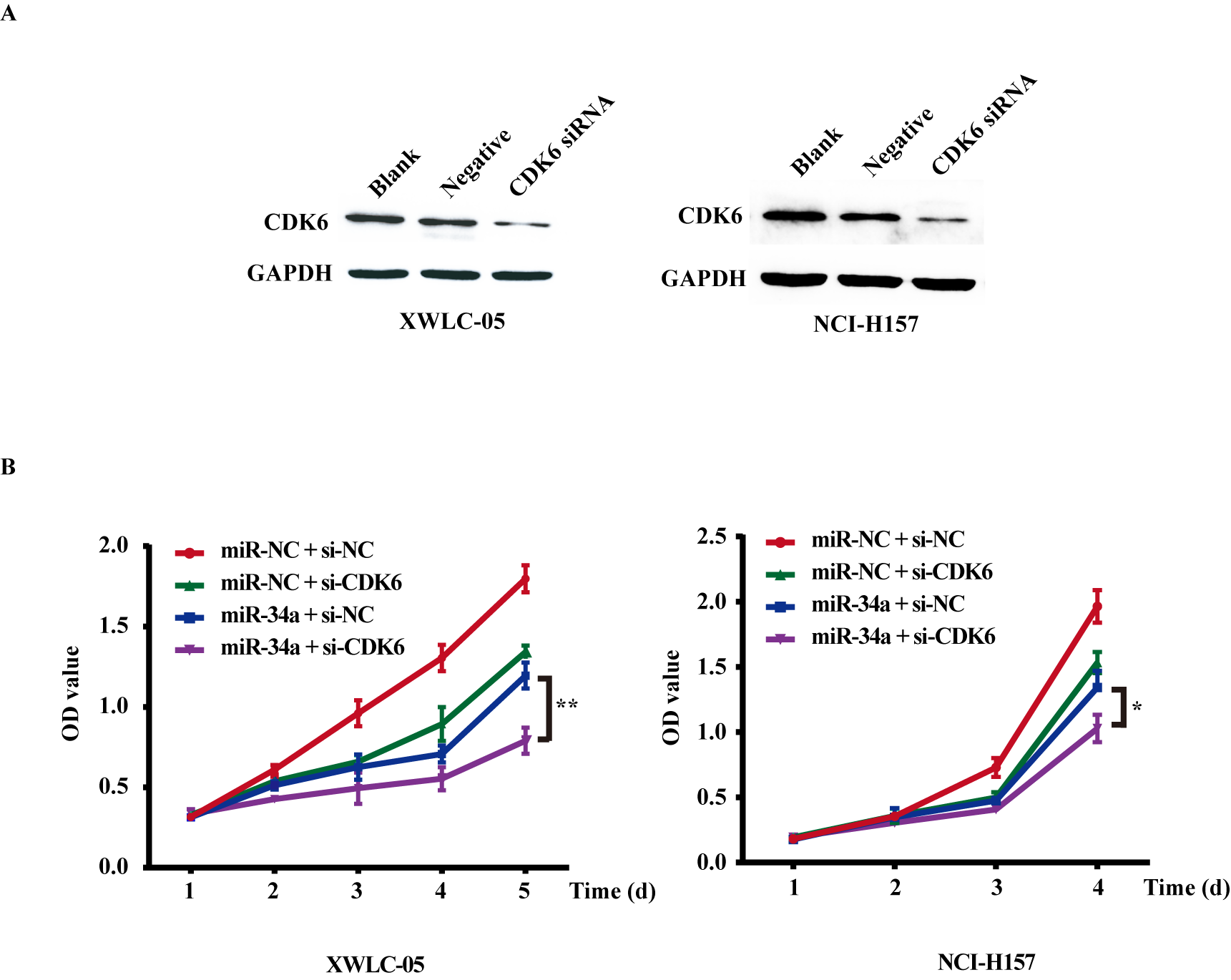

Supplement: Supplementary Figure 2 — CDK6 is involved in miR-34a-mediated inhibitory effect on lung cancer cell growth. (A) XWLC-05 and NCI-H157 cells were transfected with CDK6 siRNA, or scramble siRNA at a final concentration of 50 nM using Lipofectamine 2000 (Invitrogen). The levels of CDK6 protein expression were measured by western blot. GAPDH was used as the internal control. Blank: XWLC-05 and NCI-H157 cells; Negative: XWLC-05 and NCI-H157 cells transfected with scramble siRNA. (B) CCK8 assay was used to detect the viability of the cells co-transfected with si-CDK6 and miR-34a. [file Image_2.tif]
